# Left paraduodenal hernia: A rare cause of acute abdomen

**DOI:** 10.11604/pamj.2014.17.230.3546

**Published:** 2014-03-27

**Authors:** Karim Ibn Majdoub Hassani, Younes Aggouri, Said Ait laalim, Imane Toughrai, Khalid Mazaz

**Affiliations:** 1 Faculté de Médecine et de Pharmacie de Fès, Université Sidi Mohammed Ben Abdellah, Département de Chirurgie, CHU Hassan II, Fès, Maroc

**Keywords:** Paraduodenal hernia, left, acute abdomen

## Abstract

Paraduodenal hernia is a rare congenital anomaly that arises from an error of rotation of the midgut. The duodenum and the small intestine become trapped in a sac which is lined by the peritoneum, behind the mesentery of the colon, either to the right or left of the midline. It is therfore a rare and potentially life-threatening condition that can cause intestinal obstruction progressing to strangulation and perforation. We report a case of a 55-year-old patient presenting a left paraduodenal hernia diagnosed intraoperatively after being operated on in the emergency setting for acute abdomen. The small bowel was twisted upon its mesentery and was entrapped in a large left paraduodenal space. Fortunately, once the bowel was reduced from the paraduodenal space, the blood flow was reestablished and the small bowel resumed a proper functioning. The mouth of the sac was obliterated by suture opposition to the posterior wall. The patient's subsequent hospital course was uneventful, and he was discharged in satisfactory condition 4 days postoperatively.

## Introduction

Paraduodenal hernias are rare types of hernias which result from incomplete rotation of the midgut. They may cause acute abdominal pain, chronic digestive disorders, and nonspecific or mild symptoms such as nausea and vomiting. Therefore, because of its highly variable symptoms and signs, preoperative diagnosis of paraduodenal hernia is not always possible. This may be an incidental discovery at laparotomy or a rare cause of small bowel obstruction progressing to strangulation and perforation. Timely surgical intervention minimizes the mortality and morbidity associated with the acute presentation of this hernia.

## Patient and observation

**Figure 1:**
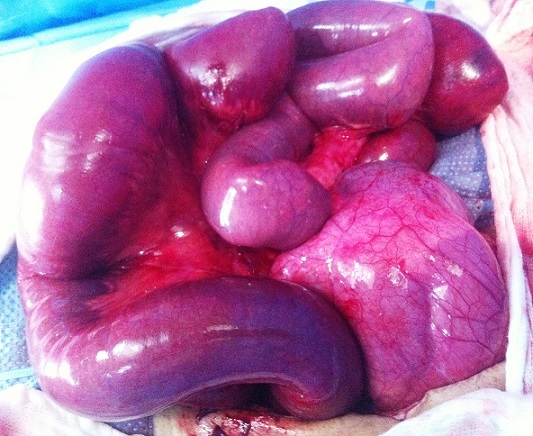
Small gut in the left paraduodenal area enclosed in a hernia sac

**Figure 2:**
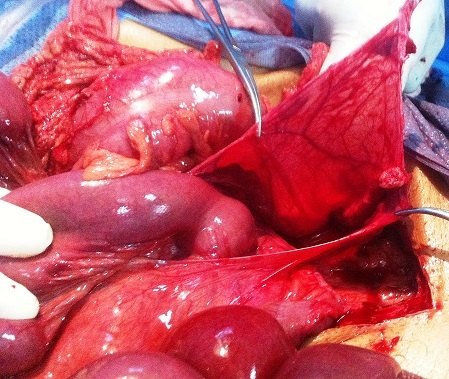
Paraduodenal hernia sac opened and the bowel reduced from it

A 55-year-old patient was presented to the emergency department of our hospital after several hours of severe and constant diffuse abdominal pain. There were no relieving or instigating factors. The pain was so sharp and was followed by non bilious emesis consistent with gastric contents. The pqtient denied any history of weight loss, chronic abdominal pain, or other gastrointestinal symptoms. He was previously asymptomatic with no history of abdominal surgery. On physical examination, the patient was moderately dehydrated with mild tachycardia (96 pulse / min) but had normal blood pressure. The abdominal examination showed signs of peritoneal irritation. The patient had generalized tenderness with epigastric and periumbelical predominance. Laboratory studies were significant for an elevated white blood cell count of 16,400 with a left shift on differential smear. Plain abdominal radiograph showed small fluid levels within bowel walls. The patient was thus taken to the operating room urgently for exploratory laparotomy, which showed dusky but viable small bowel, twisted upon its mesentery and entrapped in a large left paraduodenal space Figure 1. Once the bowel was reduced from the paraduodenal space and the volvulus was untwisted, the blood flow was reestablished and the small bowel resumed a healthy appearance Figure 2. Upon inspection, the position of the ligament of Trietz was normal. In addition, there were no other signs of malrotation. The paraduodenal space was closed by approximating the mesocolon to the base of the mesentery, with care taken not to injure the inferior mesenteric vein. The patient's subsequent hospital course was uneventful. He was discharged in satisfactory condition 4 days postoperatively.

## Discussion

**Figure 3:**
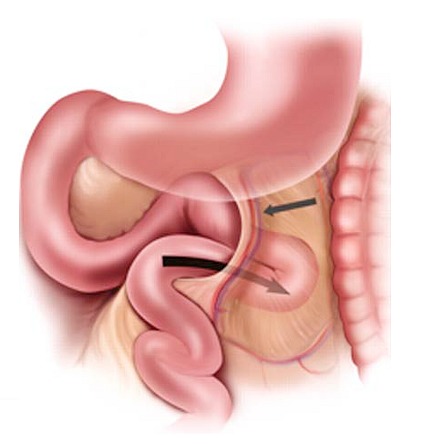
Illustration showing a loop of small bowel prolapsing (curved arrow) through Landzert's fossa, located behind the inferior mesenteric vein and left colic artery (straight arrow)

Internal hernias are uncommon cause of intestinal obstruction and occur when abdominal contents are trapped within a compartment of the abdominal cavity. It is a rare condition with an incidence of < 1% of all cases of bowel obstruction and up to 5.8% of all cases of small bowel obstruction [1–3]. The overall male/female sex ratio for internal hernia is approximately three [4]. Paraduodenal hernia constitutes 53% of all cases of internal hernias, of which 40% and 13% are of left and right paraduodenal hernias, respectively [5, 6]. This condition involves the protrusion of a viscus through a peritoneal or mesenteric opening [2]. There are many controversies and theories regarding the exact origin of paraduodenal hernias. However, the most widely accepted theory is that they result from an error in intestinal rotation and fixation that leads to entrapment of the small bowel between the mesocolon and the posterior abdominal wall. Right and left paraduodenal hernias are separate entities, differing in anatomic position and also in embryologic origin, as well [7]. They are characterized by abnormal fixation of the duodenum and jejunum. Left paraduodenal hernias are congenital anomalies formed during midgut rotation, when the small bowel invaginates into an avascular segment of the left mesocolon. The small bowel becomes entrapped between the mesocolon and the posterior abdominal wall, forming the anterior wall of the hernia sac. It has therefore been proposed that a more appropriate name for a paraduodenal hernia may be a congenital ‘mesocolic’ hernia [8]. The space into which the bowel herniates is called Landzert's fossa, and is found behind the fourth part of the duodenum Figure 3 [9]. At autopsy Landzert's fossa has been found to be present in approximately 2% of the population [10, 11]. Right paraduodenal hernias are also congenital in origin. They arise when the bowel herniates through a defect in the firstbpart of the jejunal mesentery called Waldeyer's fossa. It's found in 1% of the populationbat autopsy [12]. The hernia is found in the right side of the transverse mesocolon and extends inferolaterally behind the ascending mesocolon. Right-sided paraduodenal hernias are usually larger and more fixed than in left-sided paraduodenal hernias [13]. They are in addition associated with the small bowel non-rotation. The natural history of this type of internal hernia is to remain asymptomatic during the lifetime of a person [14]. Although it is not high on the list of differential diagnosis, there is however a 50% lifetime risks of obstruction when a left paraduodenal hernia is present [15, 16]. The symptoms and signs of aleft paraduodenal hernia range from minor to severe and may include nausea, vomiting, nonspecific abdominal pain, bowel obstruction, and peritonitis [17, 3]. Clinically, most patients present ill-defined episodes of abdominal pain, often progressing to partial or complete intestinal obstruction [18, 19]. Paraduodenal hernia presents dramatically, causes a non-specific clinical picture and often reduces spontaneously, leading to diagnostic difficulties. Although it is a rare cause of intestinal obstruction, it has a high mortality [20]. The overall mortality rate is 20%, and the mortality rate is up to 50% and 100% in the case of treated and untreated strangulated bowel or ischemic bowel, respectively [2, 3, 5].

Patients with a left paraduodenal hernia usually present during the fourth to sixth decade of life (which is the case of our patient), the mean age of presentation is 38.5 years [20]. On X-ray, paraduodenal hernias were classically described as a clustering of small-bowel loops in the upper right or left quadrants. CT has become the method of choice in diagnosing any internal hernia. In typical CT images, left paraduodenal hernia shows a cluster of dilated bowel segments with engorged and displaced mesenteric vessels at the hernial orifice [21]. If small bowel obstruction is not present, the collapsed bowel loops may be mistaken for a soft tissue mass. A high index of suspicion for this condition can help avoid unnecessary and unsuitable invasive procedures such as CT-guided biopsy [19]. Barium-enhanced studies such as upper gastrointestinal series, abdominal ultrasonography, and angiography are other diagnostic imaging modalities that can be used. Of these, barium-enhanced upper gastrointestinal series and CT are more useful for assessing internal hernia [21]. As in our case, left paraduodenal hernia can be diagnosed preoperatively in an emergency surgery for an occlusion or a surgical abdomen. Operative repair involves reduction of the hernia contents and closure of the paraduodenal defect. Occasionally, the paraduodenal defect may need to be enlarged to reduce the engorged loops of the bowel. Incising the mesocolon through an avascular section distal to the lower edge of the paraduodenal defect avoids injury of vessels in the mesocolon [22]. Once the incarcerated small bowel is reduced, the defect must be closed while being careful not to injure the adjacent mesenteric vessels, particularly the inferior mesenteric vein. Paraduodenal hernias are associated with intestinal ischemia in 20% of the cases, which is partly attributable to the difficulty in achieving a diagnosis, with resultant delay in treatment. The reported 20% mortality rate also reflects the problems with delayed therapy. As for incidentally noted paraduodenal defects, the 50% reported lifetime risk of incarceration mandates that it be repaired [23]. While an open surgery is the usual approach to this condition, successful laparoscopic repair of the right [24] and left [25, 26] paraduodenal hernias have been reported in the literature. A recent small case series comparing laparoscopic to open repair of paraduodenal hernias showed that the laparoscopic approach resulted in a shorter hospital stay, earlier intake of soft diet and a lower rate of postoperative ileus [26].

## Conclusion

Paraduodenal hernia is a relatively rare cause of acute abdomen. Therefore, its diagnosis is often incorrect or delayed owing to its variable clinical manifestations. Although this congenital anomaly is uncommon, it should be taken into consideration in the differential diagnosis of any patient with small bowel obstruction in the absence of previous abdominal surgery. It is thus important for a medical practitioner to recognize it in order to start appropriate treatment without a delay.

## References

[1] Davis R (1975). Surgery of left paraduodenal hernia. Am J Surg..

[2] Ghahremani GG, Gore RM, Levine MS (2000). Abdominal and pelvic hernias. Textbook of gastrointestinal radiology.

[3] Newsom BD, Kukora JS (1986). Congenital and acquired internal hernias: unusual causes of small bowel obstruction. Am J Surg..

[4] Fan HP, Yang AD, Chang YJ, Juan CW, Wu HP (2008). Clinical spectrum of internal hernia: a surgical emergency. Surg Today..

[5] Meyers MA (2000). Dynamic radiology of the abdomen: normal and pathologic anatomy.

[6] Dritsas ER, Ruiz OR, Kennedy GM, Blackford J, Hasl D (2001). Paraduodenal hernia: a report of two cases. Am Surg..

[7] Shinohara T, Okugawa K, Furuta C (2004). Volvulus of the small intestine caused by right paraduodenal hernia: a case report. J Pediatr Surg..

[8] Willwerth BM, Zollinger RM, Izant RJ (1974). Congenital mesocolic (paraduodenal) hernia, embryologic basis of repair. Am J Surg..

[9] Falk GA, Yurcisin BJ, Sell HS (2010). Left paraduodenal hernia: case report and review of the literature. BMJ Case Reports.

[10] Blachar A, Federle MP (2002). Internal hernia: an increasingly common cause of small bowel obstruction. Semin Ultrasound CT MR..

[11] Osadchy A, Weisenberg N, Wiener Y (2005). Small bowel obstruction related to left-side paraduodenal hernia: CT findings. Abdom Imaging..

[12] Selùuk D, Kantarci F, Ogüt G (2005). Radiological evaluation of internal abdominal hernias. Turk J Gastroenterol..

[13] Martin LC, Merkle EM, Thompson WM (2006). Review of internal hernias: radiographic and clinical findings. AJR Am J Roentgenol..

[14] Manji R, Warnock GL (2001). Left paraduodenal hernia: an unusual cause of small bowel obstruction. Can J Surg..

[15] Brigham RA, Fallon WF, Saunders JR, Harmon JW, d'Avis JC (1984). Paraduodenal hernia: diagnosis and surgical management. Surgery..

[16] Palanivelu C, Rangarajan M, Jategaonkar PA, Anand NV, Senthilkumar K (2008). Laparoscopic management of paraduodenal hernias: mesh and mesh-less repairs, a report of four cases. Hernia..

[17] Yoo HY, Mergelas J, Seibert DG (2000). Paraduodenal hernia: a treatable cause of upper gastrointestinal tract symptoms. J Clin Gastroenterol..

[18] Brigham RA, d'Avis JC, Nyhus LM, Condon RE (1989). Paraduodenal hernia. Hernia.

[19] Huang YM, Chou AB, Wu YK, Wu CC, Lee MC, Chen HT (2005). Left paraduodenal hernia presenting as recurrent small bowel obstruction. World J Gastroenterol.

[20] Khan MA, Lo AY, Vande Maele DM (1998). Paraduodenal hernia. Am Surg..

[21] Blachar A, Federle MP, Brancatelli G, Peterson MS, Oliver JH, Li W (2001). Radiologist performance in the diagnosis of internal hernia by using specific CT findings with emphasis on transmesenteric hernia. Radiology..

[22] Manji R, Warnock GL (2001). Left paraduodenal hernia: an unusual cause of small-bowel obastruction. Can J Surg..

[23] Kurachi K, Nakamura T, Hayashi T (2006). Left paraduodenal hernia in an adult complicated by ascending colon cancer: a case report. World J Gastroenterol..

[24] Antedomenico E, Singh NN, Zagorski SM (2004). Laparoscopic repair of a right paraduodenal hernia. Surg Endosc..

[25] Uematsu T, Kitamura H, Iwase M (1998). Laparoscopic repair of a paraduodenal hernia. Surg Endosc..

[26] Jeong GA, Cho GS, Kim HC (2008). Laparoscopic repair of paraduodenal hernia: comparison with conventional open repair. Surg Laparosc Endosc Percutan Tech..

